# Cold Stress and Nitrogen Deficiency Affected Protein Expression of Psychrotrophic *Dyadobacter psychrophilus* B2 and *Pseudomonas jessenii* MP1

**DOI:** 10.3389/fmicb.2017.00430

**Published:** 2017-03-14

**Authors:** Deep C. Suyal, Saurabh Kumar, Amit Yadav, Yogesh Shouche, Reeta Goel

**Affiliations:** ^1^Department of Microbiology, College of Basic Sciences and Humanities, G. B. Pant University of Agriculture and TechnologyPantnagar, India; ^2^Microbial Culture Collection, National Centre for Cell Science, Pune University CampusPune, India

**Keywords:** differential proteomics, Western Indian Himalaya, nitrogen fixation, cold diazotrophy, psychrotrophs, 2-D gel electrophoresis

## Abstract

Nitrogen (N) deficiency and low temperature conditions are the prominent facet of Western Himalayan agro-ecosystems. A slight change in the environment alters the protein expression of the microorganisms. Therefore, proteomes of the two psychrotrophs *Dyadobacter psychrophilus* B2 and *Pseudomonas jessenii* MP1 were analyzed using two dimensional electrophoresis and MALDI–TOF–MS, to determine the physiological response of altitudinally different but indigenous microorganisms in response to cold stress under N depleting conditions. Functional assessment of 150 differentially expressed proteins from both the psychrotrophs revealed several mechanisms might be involved in cold stress adaptation, protein synthesis/modifications, energy metabolism, cell growth/maintenance, etc. In both the proteomes, abundance of the proteins related to energy production and stress were significantly increased while, proteins related to biosynthesis and energy consuming processes decreased. *ATP synthase subunit alpha, beta, ATP-dependent Clp protease, Enolase, groL HtpG* and *N(2)-fixation sustaining protein CowN* proteins were found to be expressed in both B2 and MP1, similarly to previously studied diazotrophs under low temperature N_2_ fixing conditions and therefore, can be considered as a biomarker for monitoring the nitrogen fixation in cold niches. Nevertheless, in both the diazotrophs, a good fraction of the proteins were related to hypothetical proteins which are still uncharacterized, thereby, suggesting the need for in-depth studies on cold adapted diazotrophs and their adaptive mechanisms.

## Introduction

Microorganisms need to adapt constantly to different environmental changes *viz*. availability of nutrients and oxygen, osmotic stress and temperature changes. To survive these conditions bacteria need to develop unique survival strategies enabling them to persist in the environment until stress is alleviated. Nitrogen depletion at low temperature creates environmental stress as well as oligotrophic conditions along with oxidative stress which is reported to be induced by cold and therefore, impose multiple stress conditions (MSC) on the microorganisms. Very few reports are available on bacterial adaptive responses under oligotrophic and low temperature conditions including *Rhodococcus biphenylivorans* (Su et al., 2015) which was found to enter the viable but non-culturable state (VBNC) state under oligotrophic and low temperature conditions. This behavior is also very common in natural environments in which bacteria remain alive with slight metabolic modifications but are difficult to be cultured in lab. Moreover, bacteria can alter their protein expression to thrive under MSC as revealed by differential proteomic analysis of psychrophilic diazotroph *Pseudomonas migulae* S10724 (Suyal et al., 2014) and psychrotrophic diazotroph *P. palleroniana* N26 (Soni et al., 2015). However, multiple studies are needed to unravel the untouched facet of multiple stress biology induced by cold stress N depleting conditions as many key issues are still unanswered. In this context, differential proteomic analysis of psychrotrophic diazotrophs *Dyadobacter psychrophilus* B2 and *Pseudomonas jessenii* MP1 strain was carried out using two dimensional gel electrophoresis (2-DE) and MALDI–TOF–MS. This study can be explored for identifying the novel proteins/peptides and/or associated biomarkers.

## Materials and Methods

### Bacterial Strain and Growth Conditions

*Dyadobacter psychrophilus* B2 (JX233788) and *P. jessenii* MP1 (JX310329) were originally isolated from the agriculture field from WIH hill, Bhowali (1654 m; 29.22°N, 79.31°E) and Munsyari (2200 m; 30.60°N, 80.20°E). Both were grown aerobically in Burk medium (Rennie, 1981; Soni et al., 2015) at 28°C. Further, both the cultures were investigated for their growth in nitrogen deficient medium at 10°C followed by *nif*H gene amplification as described earlier (Suyal et al., 2014). Bacterium was identified using 16S rDNA sequencing as described previously (Suyal et al., 2014).

### Protein Extraction, Two Dimensional Gel Electrophoresis and Gel Image Analysis

Bacterial proteins were extracted at mid log phase as described earlier (Soni et al., 2015) (Supplementary Material). Lyophilized protein samples were sent to Sandor Proteomics Pvt. Ltd., Hyderabad for 2-DE analysis. *In silico* study of 2-DE gel was carried out as reported earlier (Suyal et al., 2014; Soni et al., 2015) (Supplementary Material). The experiment was performed in triplicates.

### MALDI–TOF–MS Analysis and MASCOT Database Searches

MALDI–TOF analysis was done at Sandor Proteomics Pvt. Ltd., Hyderabad as per the previous studies (Soni et al., 2015). The data that were obtained were used in the determination of the identity of the proteins using the Mascot search tool^1^.

## Results

### Differential Proteomics of Psychrotrophs *D. psychrophilus* B2 and *P. jessenii* MP1 Strain in Response to Cold Stress Nitrogen Depleting Conditions

**FIGURE 1:**
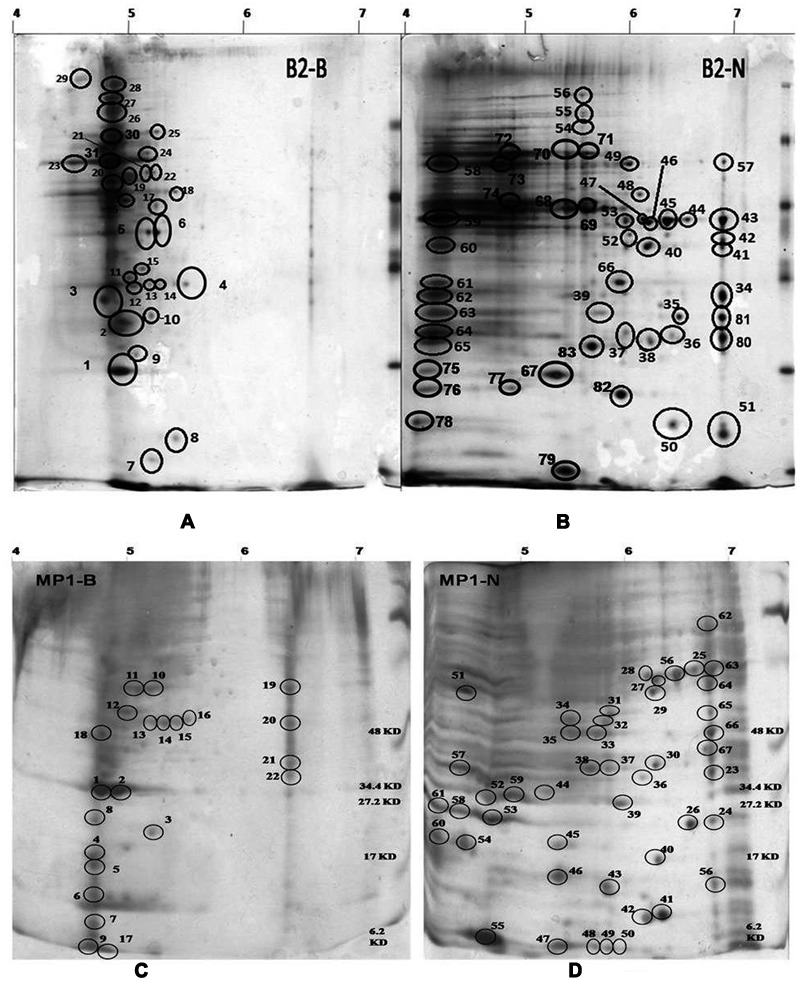
**2D profile of *Dyadobacter psychrophilus* B2 and *Pseudomonas jessenii* MP1 cultured in the absence (A**, B2-B and **C**, MP1-B; respectively) and in the presence of external nitrogen source (**B**, B2-N and **D**, MP1-N; respectively) in growth medium. Cells were harvested at mid-logarithmic phase.

Both, B2 and MP1 strains showed luxuriant growth on N deficient Burk medium, indicating their ability to fix atmospheric N_2_. Moreover, both were positive for *nif*H amplification too. *nif* genes are often used as a biomarker in diazotroph’s identification (Suyal et al., 2014; Soni et al., 2015). 2-DE was used to compare the protein expression patterns of both the bacteria B2 and MP1 separately, under two different conditions – low temperature nitrogen supplemented medium (NSM) and low temperature nitrogen deficient medium (NFM) (**Figure 1**). A pH gradient from 4 to 7 was used to analyze bacterial protein expression under NSM and NFM. A total of 82 protein spots were differentially expressed in B2, out of which 31 were found to be upregulated, while 51 were downregulated under NFM (**Figures 1A,B**). Similarly, in case of MP1, 22 proteins were upregulated while, 46 proteins were found to downregulate under NFM (**Figures 1C,D**).

A total of 12 randomly selected protein spots (6 from each bacterium) were analyzed through MALDI–TOF–MS analysis based on their expression level and molecular weight. Remaining protein spots were analyzed by analyzing 2D gel images manually (Jain et al., 2010; Suyal et al., 2014). All the spots are summarized below with their pI and Mw (Supplementary Table SM 1).

### Functional Assessment of Identified Protein Spots

**FIGURE 2:**
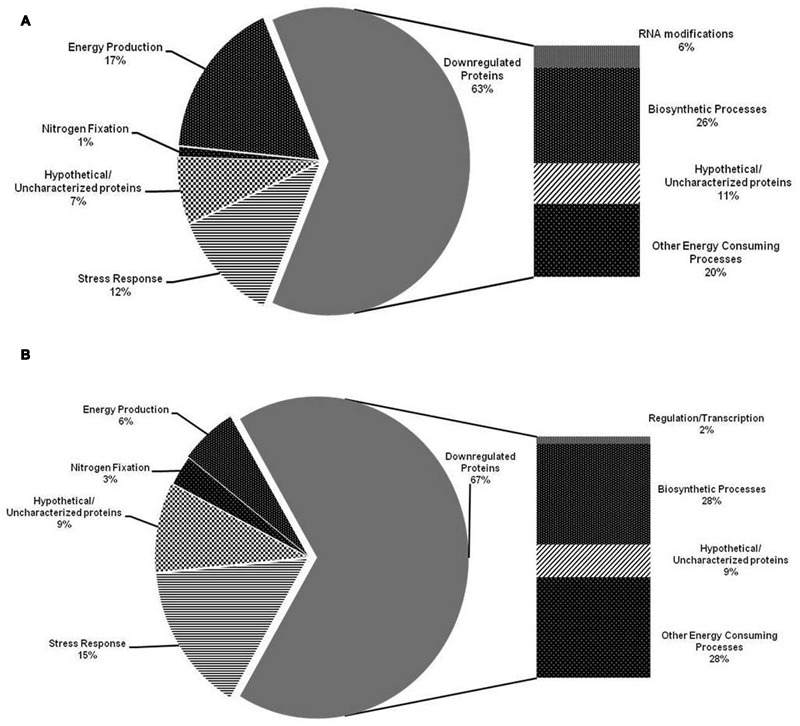
**Distribution pattern of the identified proteins upregulated and downregulated in *Dyadobacter psychrophilus* B2 (A)** and *Pseudomonas jessenii* MP1 **(B)** under low temperature N_2_ depleting conditions, according to their biological function.

A closer look at the differentially expressed proteins in psychrotolerant B2 strain indicates that major fraction of upregulated proteins was related to energy production (17%) (**Figure 2A**) *viz*. *ATP synthase subunit c, subunit E, ATP-dependent Clp protease ATP-binding subunit ClpX, ATP synthase subunit alpha, beta, Enolase* followed by stress response (12%) *viz*. *60 kDa chaperonin, Chaperone protein HtpG, Chaperone protein ClpB*. However, in case of MP1 strain stress response related proteins (15%) *viz. Chaperone protein DnaK, Chaperone protein HscA homolog, 60 kDa chaperonin, Chaperone protein TorD* were more expressed than proteins related to energy production (6%) *viz. ATP synthase subunit alpha, beta, Enolase* (**Figure 2B**). Furthermore, in both B2 and MP1 strains, nitrogen fixation related proteins (1 and 3%, respectively) were also found to upregulate *viz. N(2)-fixation sustaining protein CowN, Iron-sulfur cluster repair protein YtfE*, and *Ferredoxin-like protein in nif region*. These proteins may assist the bacteria to fix the atmospheric N through diverse strategies. Furthermore, MALDI-TOF based identification of 12 randomly selected protein spots encountered several important proteins *viz. Protein mrp homolog, Protein SlyX homolog, Aspartate carbamoyltransferase, Probable NADP-dependent dehydrogenase, Tail Sheath protein* and *Enolase* in B2 and *2-octaprenyl-6-methoxyphenyl hydroxylase, Phenylalanyl-tRNA synthetase alpha chain, Glycine cleavage H-protein, Dephospho-CoA kinase* as well as two uncharacterized proteins *UPF0260 protein ycgN* and *hypothetical protein SSON_1170* in MP1 subsequently.

**FIGURE 3:**
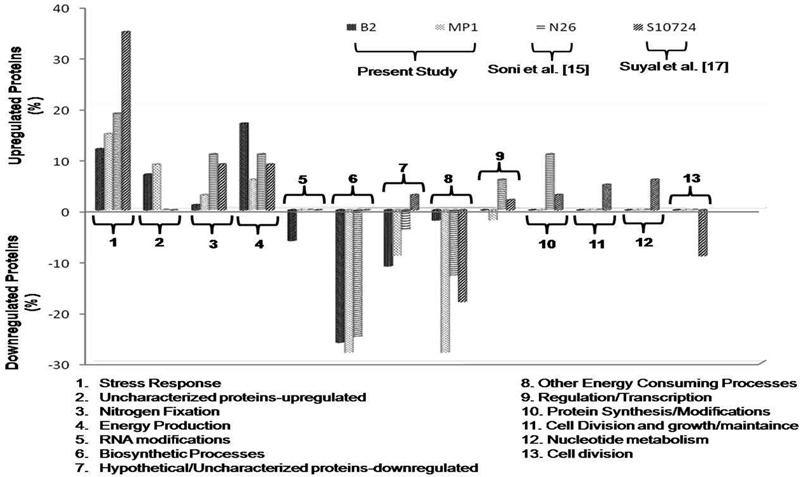
**Comparison of the identified proteins in *Dyadobacter psychrophilus* B2 and *Pseudomonas jessenii* MP1 with that of *P. palleroniana* N26 (Soni et al., 2015) and *P. migulae* S10724 (Suyal et al., 2014) under low temperature N depleting conditions, according to their biological function**.

Among the downregulated proteins, B2 and MP1 showed similar distribution pattern of the proteins related to biosynthesis (26 and 28%, respectively) and energy consuming processes (20 and 28%, respectively) (**Figure 3**). Nonetheless, B2 showed the downregulation of the proteins related to RNA modifications *viz. tRNA 2-thiocytidine biosynthesis protein TtcA* while, MP1 downregulated the expression of the proteins related to gene regulation/transcription *viz. N utilization substance protein B homolog* under NFM. Nevertheless, in both the cases, a good fraction of the upregulated proteins (7 and 9%, respectively) as well as downregulated proteins (11 and 9%, respectively) showed no resemblance with existing database and designated as Hypothetical/Uncharacterized proteins.

## Discussion

In nature, microorganisms adopt distinct strategies to cope with N depleting conditions *viz*. diazotrophs can fix atmospheric N_2_ while other microorganisms assimilate nitrate or ammonia to fulfill their needs. Both, B2 and MP1 strains showed luxuriant growth on N deficient Burk medium and thereby indicating toward their ability to fix N_2_. Similar protein expression patterns were observed under NFM conditions with slight variation in their percent distribution. In our previous studies, differential proteomic analysis of the psychrophilic diazotroph *p. migulae* S10724 (Suyal et al., 2014) and psychrotrophic diazotroph *P. Palleroniana* N26 (Soni et al., 2015) was carried out to investigate the cold adaptive nitrogen fixation and associated mechanisms. When compared to these studies, the proteins related to stress response, nitrogen fixation, and energy production were found to be upregulated in each and every case, while energy consuming processes and biosynthetic processes were always downregulated (**Figure 3**). These proteins need a detailed investigation as they can be targeted for identifying the adaptive mechanisms of N_2_ fixation in cold habitats.

*ATP synthase subunit alpha, beta, ATP-dependent Clp protease* and *Enolase* were the common energy production related proteins upregulated under NFM. *Atp-dependent Clp protease* was the proteolytic subunit and found to involve in the stress response in *Salmonella enterica* (Thomsen et al., 2002). Moreover, it is very critical for low temperature nitrogen fixation as revealed by our previous studies (Suyal et al., 2015). The differential expression of energy related proteins indicates toward the energy requirement of organisms under cold diazotrophy conditions. N_2_ fixation is an energetically costly process in terms of reducing equivalents and ATP. Their activities may therefore, mainly be related to electron transfer reactions required to provide reducing equivalents to nitrogenase and respiratory electron transport (Varley et al., 2015).

Another aspect of cold diazotrophy was to respond the stress produced by low temperature and nitrogen deprived conditions. Under normal conditions, several stress related proteins are found to present at lower levels and contributing to cellular homoeostasis under both optimal and adverse growth conditions (Prema et al., 2009; Oh et al., 2015). *60 kDa chaperonin groL* and *chaperone protein HtpG* was commonly expressed proteins in both B2 and MP1 strains under NFM. *GroL* and *Htpg* both promote the proper assembly of misfolded polypeptides under stress conditions. *HtpG* has also been observed to enhance thermotolerance in the nitrogen fixing cyanobacteria (Jae-Sung et al., 2012). Besides them, *P. jessenii* MP1 also expressed *chaperone protein TorD* which involved in the biogenesis of *TorA* and probably favors a confirmation of the apoenzyme that is competent for acquiring the cofactor and *chaperone protein HscB* homolog which is a co-chaperone involved in the maturation of iron–sulfur cluster-containing proteins. These two chaperons was also observed in psychrophilic diazotroph *P. migulae* S10724 (Suyal et al., 2014), thereby, suggesting their crucial role under low temperature N_2_ fixing conditions.

The expression of *N(2)-fixation sustaining protein CowN* in all the cold adapted diazotrophs including B2, MP1 and previously studied N26 (Soni et al., 2015) and S10724 (Suyal et al., 2014) reveals its importance for cold diazotrophy. This protein protects the N_2_ fixation ability of the nitrogenase complex from the carbon monoxide (CO) which is supposed to increase in cold conditions (Kourtelesis et al., 2015). It has been observed that under low temperature conditions iron–sulfur clusters of nitrogenase are highly susceptible to oxidative damage (Takahashi and Tokumoto, 2002). Therefore, *iron–sulfur cluster repair protein YtfE* was observed to be overexpressed in B2 and may be involved in the repair of iron–sulfur clusters of nitrogenase damaged by cold induced oxidative stress (Vinella et al., 2013). Nevertheless, *Ferredoxin-like protein in nif region* was found to upregulate in MP1 under NFM. It was also expressed in psychrophilic *P. migulae* S10724 exclusively under low temperature nitrogen fixing conditions (Suyal et al., 2014). However, its exact role under cold diazotrophy needs to be further validated.

MALDI–TOF based identification of the expressed proteins in B2 revealed the expression of *Iron–sulfur cluster carrier protein mrp* which probably helps in the proper functioning of nitrogenase complex. Further, the upregulation of *protein SlyX homolog* and *Tail Sheath protein* under NFM is not clearly understood. They may help in protein folding and perhaps showed chaperon like activities. *Aspartate carbamoyltransferase, NADP-dependent dehydrogenase* and *Enolase* are known to catalyze the pyrimidine biosynthetic pathway, pyruvate metabolism and glycolysis, respectively (Rangeshwaran et al., 2013). These proteins might be crucial for DNA synthesis/repair and energy generation processes. In contrast to B2, MP1 upregulate the protein *2-octaprenyl-6-methoxyphenyl hydroxylase* which is crucial against oxidative stress (Ammerman et al., 2008). Moreover, *Phenylalanyl-tRNA synthetase, Glycine cleavage H-protein*, and *Dephospho-CoA kinase* were also identified which are responsible for biosynthesis processes as well in protection against oxidative stress (Fan et al., 2015).

A major fraction of the downregulated proteins in B2 and MP1 was related to energy consuming processes along with biosynthetic proteins *viz. Isocitrate dehydrogenase kinase/phosphatase, Anthranilate phosphoribosyltransferase*, and *Argininosuccinate synthase*. Moreover, the downregulation of the proteins related to cell division *viz. ZipA* and *SepF* and stress response *viz. protein GrpE* and *10 kDa chaperonin* were also in agreement with the previous studies (Soni et al., 2015). The functions of these proteins might be performed by some multipurpose proteins and/or other energetically favorable alternatives, to bring down the energy requirements of the cells so that it could be utilized for nitrogen fixation. Similarly, Karas et al. (2015) studied the dual functions of the *tDNA-binding protein* from starved cells to defend cells against multiple stresses.

## Conclusion

The experimental findings provide important clues on the bacterial adaptive response to nitrogen depleting conditions in cold which will be useful in filling knowledge gaps of associated mechanisms. The common upregulated N_2_ fixation related protein *N(2)-fixation sustaining protein CowN* needs detail study to unravel its role in cold diazotrophy. This study is a step forward to reveal different enzymes/proteins involved during low temperature nitrogen fixation. However, the adaptations to protein architecture and metabolic pathways are still not well understood, hence, in depth analysis to unlock these adaptations and regulatory mechanisms could be an active area of investigation.

## Author Contributions

DS: Design of the work, experimental work, first draft of manuscript. SK: Experimental work, analysis work. AY: Analysis, manuscript drafting. YS: Sequence analysis, provided research facilities. RG: Idea of the work, manuscript checking, provide all laboratory facilities.

## Conflict of Interest Statement

The authors declare that the research was conducted in the absence of any commercial or financial relationships that could be construed as a potential conflict of interest.

## Footnotes

1 www.matrixscience.com
