# Supplementary material for: Cold Stress and Nitrogen Deficiency Affected Protein Expression of Psychrotrophic Dyadobacter psychrophilus B2 and Pseudomonas jessenii MP1
Source: Front Microbiol. 2017 Mar 14;8:430. doi: 10.3389/fmicb.2017.00430 (PMC5348510; doi:10.3389/fmicb.2017.00430)
Supplement: Supplementary file 1 [file Data_Sheet_1.docx]

**(Supplementary Material)**

**Table SM 1.** Functional characterization of spots upregulated and downregulated during low temperature N depletion condition by *Dyadobacter* *psychrophilus* B2 and *Pseudomonas jessenii* MP1. Proteins in bold were identified using MALDI-TOF analysis using MASCOT MS/MS ion search with significant threshold p<0.05.

| **S. No.** | **PI** | **MW (KD)** | | **Annotated Species** | | | **Description** |
| --- | --- | --- | --- | --- | --- | --- | --- |
| ***Dyadobacter* *psychrophilus* B2** | | | | | | | |
| **Upregulated Proteins** | | | | | | | |
|  | **4.30** | | **15.076** | | ***Helicobacter pylori*** | **Protein mrp homolog** | |
|  | **5.09** | | **21.570** | | ***Azotobacter vinelandii*** | **Protein SlyX homolog** | |
|  | **4.90** | | **24.074** | | ***Xylella fastidiosa*** | **Aspartate carbamoyltransferase** | |
|  | **5.72** | | **25.519** | | ***Dichelobacter nodosus*** | **Probable NADP-dependent dehydrogenase** | |
|  | **5.45** | | **38.767** | | ***Pseudomonas syringae*** | **Tail Sheath protein** | |
|  | **5.33** | | **45.655** | | ***Escherichia coli*** | **Enolase** | |
|  | 5.36 | | 7.310 | | *Nitrobacterwinogradskyi (strain Nb-255 )* | UPF0434 protein Nwi_0075 | |
|  | 5.61 | | 9.5440 | | *Desulfococcusoleovorans* | ATP synthase subunit c | |
|  | 5.00 | | 14.000 | | *Azorhizobium caulinodans* | N(2)-fixation sustaining protein CowN | |
|  | 5.38 | | 21.783 | | *Metallosphaera sedula* | V-type ATP synthase subunit E | |
|  | 5.20 | | 26.305 | | *Mycobacterium smegmatis* | Uncharacterized protein MSMEG_2782/MSMEI_2713 | |
|  | 5.23 | | 24.883 | | *Escherichia coli* | Iron-sulfur cluster repair protein YtfE | |
|  | 5.38 | | 25.501 | | *Cupriavidusnecator (strain ATCC 17699 / H16)* | Ribulose-phosphate 3-epimerase, chromosomal | |
|  | 5.45 | | 24.968 | | *Corynebacterium glutamicum* | Urease accessory protein UreF | |
|  | 5.30 | | 27.921 | | *Saccharomyces cerevisiae* | Uncharacterized protein YMR134W | |
|  | 5.12 | | 47.771 | | *Marinomonas sp. (strain MWYL1)* | Probable periplasmic serine endoproteaseDegP-like | |
|  | 5.44 | | 46.230 | | *Serratiaproteamaculans (strain 568)* | ATP-dependent Clp protease ATP-binding subunit ClpX | |
|  | 5.64 | | 49.637 | | *Rickettsia canadensis* | ATP-dependent protease ATPase subunit HslU | |
|  | 5.30 | | 55.450 | | *Alteromonasmacleodii (strain DSM 17117)* | ATP synthase subunit alpha | |
|  | 5.18 | | 57.306 | | *Staphylococcus xylosus* | Sucrose-6-phosphate hydrolase | |
|  | 5.34 | | 58.282 | | *Maricaulismaris (strain MCS10)* | Phosphoenolpyruvatecarboxykinase [ATP] | |
|  | 5.38 | | 57.798 | | *Bradyrhizobium sp.* | 60 kDa chaperonin groL | |
|  | 4.59 | | 62.693 | | *Mycobacterium tuberculosis (strain CDC 1551)* | Uncharacterized PPE family protein PPE12 | |
|  | 5.33 | | 67.209 | | *Methylobacterium sp.* | Chaperone protein HtpG | |
|  | 5.44 | | 73.225 | | *Streptococcus sanguinis (strain SK36)* | DNA mismatch repair protein MutL | |
|  | 5.01 | | 87.811 | | *Mycoplasma penetrans* | ATP synthase subunit beta | |
|  | 4.95 | | 94.438 | | *Streptomyces coelicolor* | Chaperone protein ClpB | |
|  | 5.03 | | 99.887 | | *Neurospora crassa* | Plasma membrane ATPase | |
|  | 4.57 | | 102.107 | | *Corynebacterium efficiens* | Aconitate hydratase A | |
|  | 5.01 | | 86.516 | | *Desulfovibrio vulgaris subsp. vulgaris* | DNA ligase | |
|  | 5.01 | | 63.037 | | *Schizosaccharomyces pombe* | Uncharacterized protein C16C4.02c | |
| **Downregulated Proteins** | | | | | | | |
|  | 6.55 | | 20.609 | | *Photorhabdusluminescens subsp. laumondii* | UPF0115 protein plu3198 | |
|  | 6.54 | | 23.00 | | *Pseudomonas putida (strain F1 )* | Uracil phosphoribosyltransferase | |
|  | 6.54 | | 25.964 | | *Chlamydia muridarum (strain MoPn / Nigg)* | 2,3-bisphosphoglycerate-dependent phosphoglyceratemutase | |
|  | 6.18 | | 25.360 | | *Schizosaccharomycespombe (strain 972 )* | Uncharacterized protein C19F8.05 | |
|  | 5.80 | | 19.964 | | *Halorhodospira halophila* | ATP-dependent protease subunit HslV | |
|  | 5.95 | | 20.128 | | *Archaeoglobusfulgidus (strain ATCC 49558 )* | Uncharacterized protein AF_2025 | |
|  | 5.77 | | 21.364 | | *Geobacillusthermodenitrificans* | Glutamine amidotransferase subunit PdxT | |
|  | 5.75 | | 31.516 | | *Sulfolobusacidocaldarius (strain ATCC 33909)* | Uncharacterized protein Saci_1674 | |
|  | 6.53 | | 31.188 | | *Pseudomonas fluorescens (strain Pf-5 )* | tRNA 2-thiocytidine biosynthesis protein TtcA | |
|  | 6.54 | | 32.443 | | *Acidovoraxcitrulli (strain AAC00-1)* | NAD kinase | |
|  | 6.55 | | 37.121 | | *Geobacter uraniireducens* | Phosphate acyltransferase | |
|  | 6.26 | | 38.209 | | *Synechocystis sp. (strain PCC 6803 / Kazusa)* | N-acetyl-gamma-glutamyl-phosphate reductase | |
|  | 6.10 | | 36.335 | | *Neisseria meningitidisserogroup C* | UDP-3-O-acylglucosamine N-acyltransferase | |
|  | 5.91 | | 35.397 | | *Shewanellaloihica (strain ATCC BAA-1088 )* | tRNA 2-thiocytidine biosynthesis protein TtcA | |
|  | 5.97 | | 38.228 | | *Delftiaacidovorans* | Dihydroorotase | |
|  | 5.74 | | 51.009 | | [*Campylobacter jejuni subsp. jejuni*](http://www.uniprot.org/taxonomy/354242) | Bifunctional protein HldE | |
|  | 5.79 | | 64.656 | | *Escherichia coli O1:K1 / APEC* | L-fucoseisomerase | |
|  | 6.15 | | 11.139 | | *Pectobacteriumcarotovorum* | Flagellar hook-basal body complex protein FliE | |
|  | 6.55 | | 10.184 | | *Escherichia coli (strain K12)* | Uncharacterized protein YbaV | |
|  | 5.78 | | 32.968 | | *Escherichia coli O1:K1 / APEC* | Ribonuclease BN | |
|  | 5.77 | | 36.122 | | *Prosthecochlorisvibrioformis (strain DSM 265)* | Ketol-acid reductoisomerase | |
|  | 5.45 | | 82.276 | | *Mycobacterium ulcerans* | Polyphosphate kinase | |
|  | 5.42 | | 93.198 | | *Pelobacterpropionicus (strain DSM 2379)* | Leucine--tRNA ligase | |
|  | 5.41 | | 106.191 | | *Syntrophobacterfumaroxidans* | Translation initiation factor IF-2 | |
|  | 6.51 | | 68.083 | | *Pyrobaculum islandicum* | Glutamyl-tRNA(Gln) amidotransferase subunit E | |
|  | 4.34 | | 61.298 | | *Mycobacterium tuberculosis (strain CDC 1551)* | Uncharacterized PE-PGRS family protein PE_PGRS10 | |
|  | 4.30 | | 45.967 | | *Haloferax volcanii* | UDP-glucose 6-dehydrogenase AglM | |
|  | 4.34 | | 36.041 | | *Cell division protein ZipA* | Vibrio campbellii | |
|  | 4.29 | | 31.587 | | *Pseudomonas mendocina* | Ribosomal protein L11 methyltransferase | |
|  | 4.30 | | 26.446 | | *Lysinibacillussphaericus (strain C3-41)* | Adapter protein MecA | |
|  | 4.32 | | 23.192 | | *Staphylococcus carnosus (strain TM300)* | 50S ribosomal protein L25 | |
|  | 4.32 | | 20.913 | | *Cell division protein SepF* | Prochlorococcus marinus | |
|  | 4.24 | | 19.935 | | *Arthrobacteraurescens (strain TC1)* | Ribosome maturation factor RimM | |
|  | 5.91 | | 32.276 | | *Burkholderia cenocepacia* | NAD kinase | |
|  | 5.22 | | 17.074 | | *Aeromonas hydrophila* | Arginine repressor | |
|  | 5.38 | | 34.931 | | *Arthrobacter sp.* | N-acetyl-gamma-glutamyl-phosphate reductase | |
|  | 5.52 | | 34.823 | | *Burkholderia mallei (strain SAVP1)* | 3-oxoacyl-[acyl-carrier-protein] synthase 3 | |
|  | 5.39 | | 38.732 | | *Biotin synthase* | Shewanella sp. (strain ANA-3) | |
|  | 5.52 | | 46.522 | | *Azoarcus sp.* | ATP-dependent Clp protease ATP-binding subunit ClpX | |
|  | 4.80 | | 44.826 | | *Clavibacter michiganensis* | Argininosuccinate synthase | |
|  | 4.79 | | 42.385 | | *Nocardioides sp.* | S-adenosylmethionine synthase | |
|  | 4.80 | | 34.300 | | *Listeria welshimeri serovar 6b* | Glycine--tRNA ligase alpha subunit | |
|  | 4.13 | | 17.343 | | *Yersinia enterocolitica* | Regulator of ribonuclease activity A | |
|  | 4.18 | | 17.145 | | *Aeromonas hydrophila* | Endoribonuclease YbeY | |
|  | 5.01 | | 16.128 | | *nucleotidohydrolase* | Shewanella sp. (strain ANA-3) | |
|  | 4.01 | | 13.743 | | *Halobacterium salinarum* | 30S ribosomal protein S6e | |
|  | 5.20 | | 6.630 | | *Pseudomonas fluorescens* | UPF0434 protein PFLU_3771 | |
|  | 6.46 | | 21.431 | | *Lactobacillus reuteri* | Protein GrpE | |
|  | 6.70 | | 24.65 | | *Escherichia coli* | Putative uncharacterized protein YmjD | |
|  | 5.71 | | 14.452 | | *Bacillus subtilis (strain 168)* | Uncharacterized protein YbfJ | |
|  | 5.49 | | 20.154 | | *Campylobacter fetus subsp. fetus* | Ribosome maturation factor RimM | |
| ***Pseudomonas jessenii* MP1** | | | | | | | |
| **Upregulated Proteins** | | | | | | | |
|  | **4.80** | | **45.654** | | ***Vibrio sp. 16*** | **2-octaprenyl-6-methoxyphenyl hydroxylase** | |
|  | **5.00** | | **40.966** | | ***Desulfovibrio vulgaris*** | **Phenylalanyl-tRNA synthetase alpha chain** | |
|  | **5.11** | | **25.280** | | ***Solibacter usitatus Ellin6076*** | **Glycine cleavage H-protein** | |
|  | **4.30** | | **16.867** | | ***Bacillus cereus*** | **Dephospho-CoA kinase** | |
|  | **4.30** | | **15.076** | | ***Escherichia coli*** | **UPF0260 protein ycgN** | |
|  | **4.30** | | **11.972** | | ***Shigella sonnei*** | **hypothetical protein SSON_1170** | |
|  | 4.70 | | 13.237 | | [*Azoarcus sp.*](http://www.uniprot.org/taxonomy/62928) | N(2)-fixation sustaining protein CowN | |
|  | 4.63 | | 24.813 | | [*Shewanella pealeana*](http://www.uniprot.org/taxonomy/398579) | Chaperone protein TorD | |
|  | 4.65 | | 68.77 | | [*Acidovorax sp.*](http://www.uniprot.org/taxonomy/232721) | UPF0434 protein Ajs_2306 | |
|  | 5.33 | | 67.209 | | *Methylobacterium sp.* | Chaperone protein HtpG | |
|  | 5.12 | | 65.688 | | [*Rickettsia akari*](http://www.uniprot.org/taxonomy/293614) | Chaperone protein HscA homolog | |
|  | 5.00 | | 57.820 | | [*Synechococcus sp.*](http://www.uniprot.org/taxonomy/316278) | 60 kDa chaperonin groL | |
|  | 5.17 | | 55.626 | | *Marinobacter hydrocarbonoclasticus* | ATP synthase subunit beta | |
|  | 5.25 | | 54.867 | | [*Bacillus thuringiensis*](http://www.uniprot.org/taxonomy/412694) | Probable glycine dehydrogenase [decarboxylating] subunit 2 | |
|  | 5.31 | | 55.148 | | *Shewanella sp.* | ATP synthase subunit alpha | |
|  | 5.52 | | 56.317 | | [*Methanocaldococcus jannaschii*](http://www.uniprot.org/taxonomy/243232) | UPF0288 protein MJ1412 | |
|  | 4.90 | | 6.950 | | [*Rhizobium sp.*](http://www.uniprot.org/taxonomy/394) | Ferredoxin-like protein in *nif* region | |
|  | 4.70 | | 46.473 | | *Listeria welshimeri serovar 6b* | Enolase | |
|  | 6.46 | | 67.575 | | *Enterobacter sp.* | DNA mismatch repair protein MutL | |
|  | 6.44 | | 45.170 | | *Nocardioides sp.* | DNA replication and repair protein RecF | |
|  | 6.40 | | 40.023 | | *Bacillus cereus* | DNA integrity scanning protein DisA | |
|  | 6.40 | | 37.787 | | *Rhizobium meliloti* | Protein FixB | |
| **Downregulated proteins** | | | | | | | |
|  | 6.73 | | 38.251 | | [*Agrobacterium vitis*](http://www.uniprot.org/taxonomy/311402) | NADH-quinone oxidoreductase subunit H | |
|  | 6.74 | | 23.705 | | [*Acidovorax sp.*](http://www.uniprot.org/taxonomy/232721) | Adenylate kinase | |
|  | 6.60 | | 67.590 | | *Pyrobaculum calidifontis* | Glutamyl-tRNA(Gln) amidotransferase subunit E | |
|  | 6.55 | | 23.013 | | [*Bradyrhizobium japonicum*](http://www.uniprot.org/taxonomy/224911) | Arginine biosynthesis bifunctional protein ArgJ | |
|  | 6.22 | | 66.396 | | [*Escherichia coli*](http://www.uniprot.org/taxonomy/331111) | Glutathione-regulated potassium-efflux system protein KefB | |
|  | 6.11 | | 67.206 | | *Escherichia coli* | Isocitrate dehydrogenase kinase/phosphatase | |
|  | 6.19 | | 61.944 | | [*Heliobacterium modesticaldum*](http://www.uniprot.org/taxonomy/498761) | UPF0747 protein Helmi_24320 | |
|  | 6.21 | | 39.022 | | [*Bacillus megaterium*](http://www.uniprot.org/taxonomy/1404) | Putative cobalt-precorrin-6A synthase [deacetylating] | |
|  | 5.78 | | 54.071 | | [*Rhodobacter sphaeroides*](http://www.uniprot.org/taxonomy/349101) | Cysteine--tRNA ligase | |
|  | 5.74 | | 51.009 | | [*Campylobacter jejuni subsp. jejuni*](http://www.uniprot.org/taxonomy/354242) | Bifunctional protein HldE | |
|  | 5.67 | | 46.894 | | [*Pseudomonas mendocina*](http://www.uniprot.org/taxonomy/399739) | Adenylosuccinate synthetase | |
|  | 5.39 | | 53.248 | | [*Chlamydophila felis*](http://www.uniprot.org/taxonomy/264202) | Glutamyl-tRNA(Gln) amidotransferase subunit A | |
|  | 5.41 | | 48.009 | | [*Geobacter sp.*](http://www.uniprot.org/taxonomy/443144) | Serine--tRNA ligase | |
|  | 6.00 | | 36.595 | | [*Prochlorococcus marinus*](http://www.uniprot.org/taxonomy/167555) | Anthranilate phosphoribosyltransferase | |
|  | 5.77 | | 38.725 | | [*Salmonella paratyphi B*](http://www.uniprot.org/taxonomy/1016998) | Dihydroorotase | |
|  | 5.58 | | 36.855 | | *Yersinia enterocolitica* | Aspartate--ammonia ligase | |
|  | 5.86 | | 26.323 | | [*Pasteurella multocida*](http://www.uniprot.org/taxonomy/272843) | UPF0001 protein PM0112 | |
|  | 6.22 | | 16.498 | | [*Acinetobacter baumannii*](http://www.uniprot.org/taxonomy/400667) | 3-dehydroquinate dehydratase | |
|  | 6.26 | | 9.761 | | [*Shewanella sp.*](http://www.uniprot.org/taxonomy/94122) | UPF0250 protein Shewana3_0990 | |
|  | 6.10 | | 9.441 | | [*Treponema pallidum subsp. pallidum*](http://www.uniprot.org/taxonomy/455434) | 10 kDa chaperonin | |
|  | 5.74 | | 12.882 | | [*Legionella pneumophila*](http://www.uniprot.org/taxonomy/297245) | Putative pterin-4-alpha-carbinolamine dehydratase | |
|  | 5.20 | | 32.110 | | [*Burkholderia cenocepacia*](http://www.uniprot.org/taxonomy/331272) | Acetylglutamate kinase | |
|  | 5.28 | | 18.666 | | [*Ochrobactrum anthropi*](http://www.uniprot.org/taxonomy/439375) | N utilization substance protein B homolog | |
|  | 5.30 | | 14.523 | | [*Bacillus halodurans*](http://www.uniprot.org/taxonomy/272558) | Uncharacterized protein BH0965 | |
|  | 5.33 | | 52.184 | | [*Escherichia coli O1:K1 / APEC*](http://www.uniprot.org/taxonomy/405955) | Cysteine--tRNA ligase | |
|  | 5.58 | | 51.700 | | [*Mycobacterium ulcerans*](http://www.uniprot.org/taxonomy/362242) | Cysteine--tRNA ligase. | |
|  | 5.64 | | 52.193 | | [*Burkholderia mallei*](http://www.uniprot.org/taxonomy/320388) | Glutamate--tRNA ligase | |
|  | 5.75 | | 51.903 | | [*Mycobacterium avium*](http://www.uniprot.org/taxonomy/243243) | Cysteine--tRNA ligase | |
|  | 4.37 | | 63.404 | | [*Halorubrum lacusprofundi*](http://www.uniprot.org/taxonomy/416348) | DNA ligase | |
|  | 4.83 | | 28.352 | | [*Arthrobacter aurescens*](http://www.uniprot.org/taxonomy/290340) | Indole-3-glycerol phosphate synthase | |
|  | 4.63 | | 23.504 | | [*Shewanella pealeana*](http://www.uniprot.org/taxonomy/398579) | Ribose-5-phosphate isomerase A | |
|  | 4.37 | | 19.131 | | [*Haemophilus influenzae*](http://www.uniprot.org/taxonomy/374930) | Protein-export protein SecB | |
|  | 4.62 | | 67.36 | | [*Acidovorax citrulli*](http://www.uniprot.org/taxonomy/397945) | UPF0434 protein Aave_2563 | |
|  | 4.44 | | 69.23 | | [*Mycobacterium bovis*](http://www.uniprot.org/taxonomy/233413) | Uncharacterized protein Mb2954 | |
|  | 6.41 | | 62.551 | | *Anaeromyxobacter sp.* | Proline--tRNA ligase | |
|  | 4.34 | | 36.041 | | *Vibrio campbellii* | Cell division protein ZipA | |
|  | 4.32 | | 20.913 | | *Prochlorococcus marinus* | Cell division protein SepF | |
|  | 5.03 | | 26.166 | | *Listeria welshimeri* | Uridylate kinase | |
|  | 4.20 | | 7.355 | | *Salmonella arizonae* | Phosphatidylserine decarboxylase proenzyme | |
|  | 4.21 | | 66.200 | | *Halorubrum lacusprofundi* | Arginine--tRNA ligase | |
|  | 6.68 | | 98.320 | | [*Shewanella oneidensis*](http://www.uniprot.org/taxonomy/211586) | [Protein-PII] uridylyltransferase | |
|  | 6.71 | | 69.758 | | [*Symbiobacterium thermophilum*](http://www.uniprot.org/taxonomy/292459) | tRNA uridine 5-carboxymethylaminomethyl modification enzyme | |
|  | 6.71 | | 64.976 | | [*Lysinibacillus sphaericus*](http://www.uniprot.org/taxonomy/444177) | Sulfite reductase [NADPH] hemoprotein beta-component | |
|  | 6.70 | | 54.612 | | [*Helicobacter pylori*](http://www.uniprot.org/taxonomy/85963) | Cytosol aminopeptidase | |
|  | 6.74, | | 47.055 | | [*Cronobacter sakazakii*](http://www.uniprot.org/taxonomy/290339) | ATP-dependent RNA helicase RhlB | |
|  | 6.68 | | 44.041 | | [*Methanococcus vannielii*](http://www.uniprot.org/taxonomy/406327) | Translation initiation factor 2 subunit gamma | |

**Protein Extraction:**

Single colony of both the strains strain were inoculated in 500 ml of broth, separately and grown overnight in “Nutrient broth and Burk’s medium” representing two different conditions - low temperature nitrogen sufficient condition (NSC) and low temperature nitrogen fixing condition (NFC), respectively. Cultures were harvested at 10,000 rpm for 10 min and pellets were washed twice with Normal saline solution. Pellets were then dissolved in 4ml of 0.1M Phosphate buffer saline (chilled) and sonicated for 3 min in ice. One mM PMSF (100μl) was added to the sonicated pellets. Cell suspension was centrifuged for 45 min at 10,000 rpm. The supernatants were lyophilized and were send to Sandor Proteomics Pvt Ltd, Hyderabad for further analysis.

**Two dimensional gel electrophoresis and gel image analysis:**

2D gel electrophoresis was done at Sandor Proteomics Pvt Ltd, Hyderabad using Bio-Rad 2D gel electrophoresis unit. Protein samples were solubilised in isoelectric focusing (IEF) buffer containing 7 M urea, 2 M thio-urea, 4% (w/v) CHAPS, 1% (w/v) dithiothreitol (DTT) and 0.2% (v/v) ampholytes pH 3-10 (Bio-Rad, Hercules, US). The 17 cm immobilized pH gradient (IPG) strips (pH 3-10 or 4-7, Bio-Rad, Hercules, US) were passively rehydrated for 16 h with 300 µL of cell extract samples containing 1-2 mg of protein. IEF was performed in a Protean IEF cell system (Bio-Rad, Hercules, US) with up to 50,000 VH at a maximum voltage of 10,000 V. Strips were equilibrated for 15 min in equilibration buffer I (30%, v/v, glycerol, 6 M urea, 1% DTT, a trace of bromophenol blue) and for 15 min in equilibration buffer II (equilibration solution I with DTT replaced by 4% iodoacetamide). In the second dimension, IPG strips were run vertically onto SDS-PAGE 12% gels using PROTEAN® II xi 2D Cell (Bio-Rad, Hercules, US). Gels were stained with 0.1% Coomassie Brilliant Blue R-250 (Hi-media, US) and scanned with a computer-assisted G-800 densitometer (Bio-Rad, Hercules, US). The pI and Mw of all the protein spots were calculated by analyzing the gels manually. Computational study of these spots was carried out with the help of ExPASy software package of proteomic analysis based on pI and Mw. Swiss-Prot/TrEMBL databases were searched with the help of Tagident tool for their functional analysis.

**MALDI-TOF-MS analysis and MASCOT Database Searches**

MALDI-TOF-MS Analysis was done at Sandor Proteomics Pvt Ltd, Hyderabad using Bruker Daltonics - Ultraflex™ III Mass Spectrometer with spectra internally calibrated using trypsin auto-digestion products. The obtained peptide masses were searched on the Mascot^TM^ PeptideMass Fingerprint database (Matrix Science). The data that were obtained were used in the determination of the identity of the proteins using the Mascot search tool ([www.matrixscience.com](http://www.matrixscience.com)).

**Methodology used in the study**

**
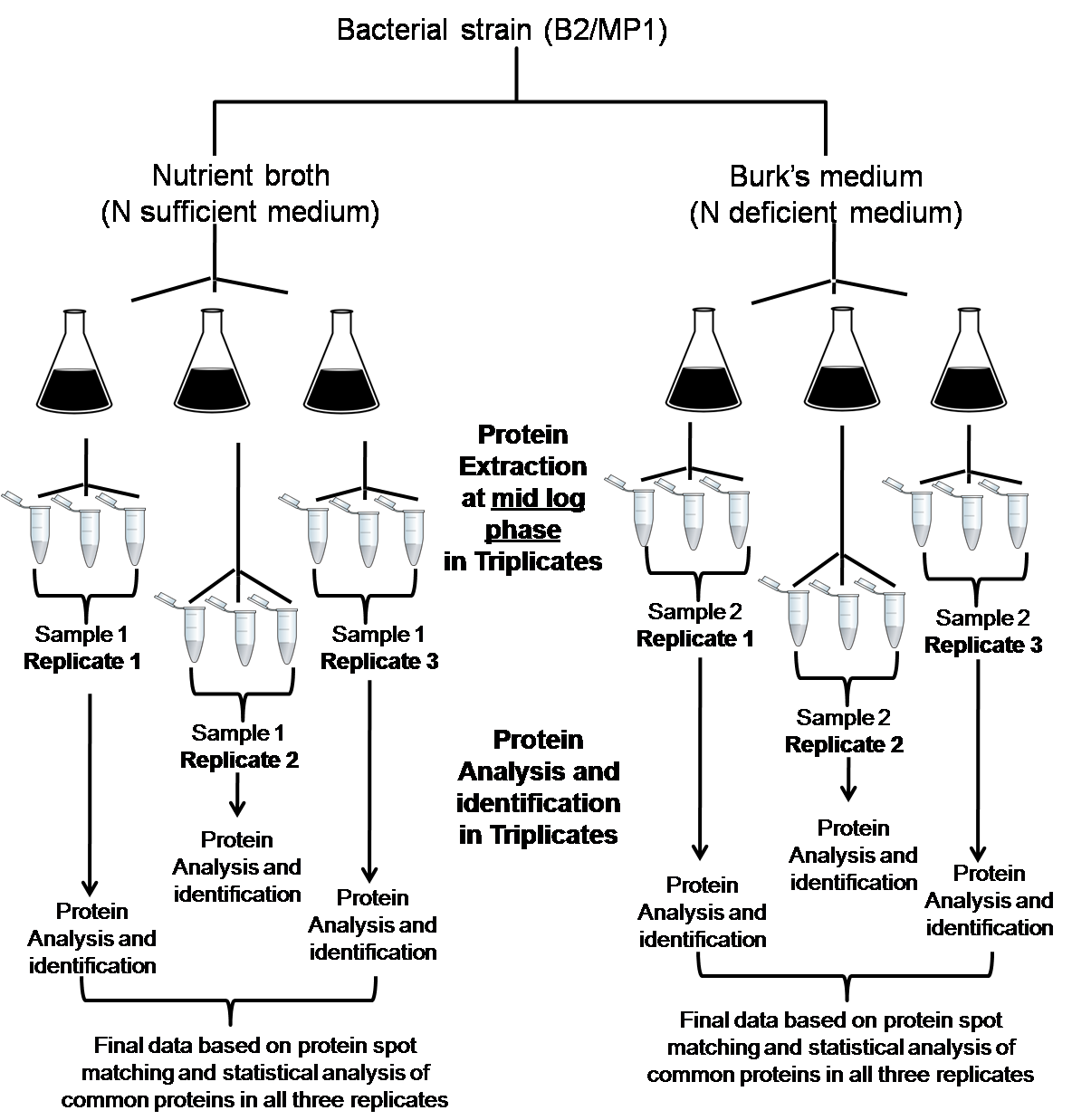
**
